# Olive Fruit Development and Ripening: Break on through to the “-Omics” Side

**DOI:** 10.3390/ijms22115806

**Published:** 2021-05-28

**Authors:** Christina Skodra, Vaia Styliani Titeli, Michail Michailidis, Christos Bazakos, Ioannis Ganopoulos, Athanassios Molassiotis, Georgia Tanou

**Affiliations:** 1 Laboratory of Pomology, Department of Horticulture, Aristotle University of Thessaloniki, 57001 Thessaloniki-Thermi, Greece; chriskod@agro.auth.gr (C.S.); vgtiteli@gmail.com (V.S.T.); msmichai@agro.auth.gr (M.M.); amolasio@agro.auth.gr (A.M.); 2 Institute of Plant Breeding and Genetic Resources, Hellenic Agricultural Organization—‘Demeter’ (ELGO-Demeter), 57001 Thessaloniki-Thermi, Greece; bazakos@ipgrb.gr (C.B.); giannis.ganopoulos@gmail.com (I.G.); 3 Institute of Soil and Water Resources, Hellenic Agricultural Organization—‘Demeter’ (ELGO-Demeter), 57001 Thessaloniki-Thermi, Greece

**Keywords:** fruit ripening, metabolomics, *Olea europaea*, olive drupe, proteomics, systems biology, transcriptomics

## Abstract

The olive tree (*Olea europaea* L. subsp. *europaea*) is the most important perennial crop in the Mediterranean region, producing table olives and oil, both appreciated for their nutraceutical value. Although olive oil quality traits have been extensively studied, much less attention has been paid to olive drupe. Olive drupe ripening is an extremely complex process involving numerous physiological and molecular changes that are unique in this fruit crop species. This review underlines the contribution of “-omics” techniques and of the recent advances in bioinformatics and analytical tools, notably next-generation sequencing and mass spectrometry, for the characterization of the olive ripening syndrome. The usage of high-dimensional datasets, such as transcriptomics, proteomics, and metabolomics, will provide a systematical description of the molecular-specific processes regulating olive fruit development and ripening. However, the incomplete sequence of the *O. europaea* L. reference genome has largely hampered the utilization of omics tools towards olive drupe research. Due to this disadvantage, the most reported -omics studies on fruit trees concern metabolomics and only a few transcriptomics and proteomics. In this review, up-to-date applications of -omics technologies towards olive drupe biology are addressed, and future perspectives in olive fruit research are highlighted.

## 1. Introduction

The double nature of *Olea europaea L.* (subsp. *europaea* var. *europaea*) as a wild element of vegetation and as a crop justifies its omnipresence in the Mediterranean Basin’s agroecosystems and as an economically significant species of the Mediterranean agriculture. This woody tree crop is being cultivated both for its oil and/or table drupes, while a few major cultivars are relevant for shaping and producing the landscape [1]. The wild form of the olive tree, formally named *Olea europaea* var *sylvestris* or oleaster, is the ancestor of the cultivated olive tree and is also considered to be one of the oldest trees worldwide [2]. Contemporary classifications recognize two taxonomic varieties of *Olea europaea*: one for wild populations (var. *sylvestris*) and one for the cultivated forms (var. *europaea*). However, it is not clear whether a secondary diversification, which comes after a single primary incident of domestication, is responsible for different olive cultivars or whether cultivated varieties are the outcome of a greater number of irrespective domestication events [3].

As a member of the Oleaceae family, *Olea europaea* L. resides in a group of plants composed of 600 species classified in 25 genera [4]. Olive trees are mainly cultivated in the Mediterranean; however, their cultivation is spread in various locations around the world that are characterized either by tropical or by mild climate conditions [5]. Particularly, olive cultivation areas in the Mediterranean basins cover a total of 12.000 hectares; that corresponds to 95% of total worldwide cultivated olive fields [3]. Except for oil, table olives consist of a product of high agricultural and nutraceutical value for global commerce. Olive trees produce fruits classified as drupes comprised by a thin, protective exocarp or epicarp; a fleshy mesocarp; and an inedible, stony endocarp surrounding the seed.

Every olive fruit tissue is characterized by unique changes in growth and development during whole fruit maturation [6]. Fruit ripening is a developmentally regulated process that involves five distinct stages, namely, (i) fruit set starting just after fertilization, (ii) seed development that mainly affects the growth of the endocarp (seed/pit) and the mesocarp development, (iii) pit hardening accompanied by oil accumulation on drupe, (iv) mesocarp development and, finally, (v) fruit maturation [7]. Ripening of olive fruits is characterized by intense changes in fruit physiology such as color alterations, derived from variations in pigment compounds (chlorophyll, flavonoids and carotenoids), texture modifications, related to changes in cell wall composition or turgor and metabolic variations concerning carbohydrate and other organic compounds production that affect organoleptic properties (nutritional value, aroma and flavor). Moreover, olive ripening is mainly orchestrated by the expression of ripening-related genes and proteins under the control of a network of metabolic pathways that are triggered by external or internal factors [8].

Understanding the molecular mechanism of the olive drupe ripening process is crucial for meeting new technological advances in olive breeding programs [9,10]. However, differences in ripening features among cultivars still cannot be sufficiently ascribable to certain genes’ expression and/or regulation. In order to elucidate this phenomenon, abundant markers along the genome, such as SNPs, are widely used [11]. Along with the constant augmentation in olive genome sequence knowledge, novel, innovative approaches in genomics and olive breeding will be provided, elucidating biological processes occurring in different tissues across various phases of fruit ripening. The evolution of novel applications, such as the genome-wide study of variants (genomics) and genes (transcriptomics) and the universal detection of proteins (proteomics) and metabolites (metabolomics), provide new insights into the olive fruit development in the “-omics” era. Genome sequencing, transcriptomic and proteomic strategies and the metabolic profile of the cultivated olive have been proved useful to identify ripening-related genes/proteins/metabolites that are contributing tremendously to a better understanding of olive drupe biology [12].

## 2. Advances in Olive Genome Assembly and Annotation and Its Significance in Population Genetic Studies

Genomics refers to the genome-wide approaches dealing with the structure, function, evolution, mapping and editing of genomes. Olive’s genome size is ~1.4 Gb and is characterized as a medium-sized plant genome [13,14]. Due to the evidence that a large fraction of the genome derived by tandem repeats amplification and Long Terminal Repeats–retrotransposons (LTR–Res) in combination with the high level of heterozygosity, the olive genome assembly at chromosome-level scaffolds is a relatively difficult task [3].

Recently, due to the enormous technological improvement of long-read sequencing platforms (PacBio and Oxford Nanopore), two more olive genome assemblies of the cultivars ‘Arbequina’ and ‘Picual’ were published [14,17]. Jimenez-Ruiz et al. (2020) studied the evolution of the olive genome during the domestication process by performing the de novo genome assembly of cv. ‘Picual,’ one of the most popular olive cultivars [17]. Rao et al. (2021) aimed to generate a new draft of cv. ‘Arbequina’ genome with sufficient quality for genome-wide studies on olive species [14]. Both studies achieved to improve the quality of the existed draft genomes. The contig N50 length was increased by 10–100 times in cv. ‘Picual’ and cv. ‘Arbequina,’ respectively, compared to oleaster genome and cv. ‘Farga’ genome (Table 1) [14,15,16,17]. The Hi-C technology was used to assemble 1.1 Gb of the cv. ‘Arbequina’ genome in 23 pseudochromosomes without the need of a previously developed genetic map as they performed with oleaster genome that only the 572 Mb were anchored in 23 pseudochromosomes [14,16]. A population genetic study was also carried out with the resequencing of 10 wild and 40 cultivated accessions, mapped on the latest draft assembly of cv. ‘Picual’ and they reported two irrespective events in olive domestication and an early genetic bottleneck. In spite of these, it was observed a high genetic diversity to the cultivated accessions, which was driven by the activation of transposable elements (TE) during the domestication process [17].

The first efforts on olive genome sequencing and assembly were carried out for *Olea europaea* L. subsp. *europaea* cv. ‘Farga’ and *Olea europaea* L. subsp. *sylvestris*. The cultivar ‘Farga’ is a Spanish olive tree about 1200 years old. It was sequenced with the whole-genome shotgun approach using Illumina short-read technology [15] (Table 1). Although, both of the genome assemblies are characterized by the high number of contigs (Table 1), the International Olive (*O. europaea*) Genome Consortium (IOGC) (http://olivegenome.org/ (accessed on 9 October 2017) managed to anchor a large number of these scaffolds to 23 pseudochromosomes through a genetic map and thus provided the first chromosome anchoring with genome-wide functional annotation of oleaster, the wild olive tree, genome [16]. Due to the fact that the wild and domesticated olive genomes have the same number of chromosomes and similar size, this genome assembly is a valuable contribution to population genetic and transcriptomic studies as a reference genome.

Currently, the Greek ‘Olive Road’ project (https://dromoielias.gr/en/ (accessed on 30 October 2018) aims to provide the de novo genome assemblies of Greek-oriented emblematic olive cultivars, such as the cv. ‘Koroneiki.’ In addition to this, through this project, we aimed to provide a large-scale transcriptomic, proteomic, metabolomic and metagenomic analysis of various Greek olive cultivars and their association with important biological phenomena, including abiotic stresses and fruit development. This approach, coupled with full-length cDNA sequencing using long-read sequencing technologies, will significantly assist in further improvement of the current olive genome functional annotation.

## 3. Application of “-Omics” Technologies to Characterize Olive Fruit Development and Ripening

### 3.1. Gene Expression of Candidate Genes Unravel Pathways Related to Olive Fruit Ripening

To achieve a closer comprehension of the genomic operation and the evolution of proper molecular tools, transcriptomic data have already been obtained for some olive genes that participate in specific functions, such as olive drupe ripening (Table 2). Particularly, understanding the regulation of evolutive patterns of fatty acid synthesis during olive development and maturation, as well as in response to several external factors, may help to build up the bases for genetic manipulation aiming at olive fruit and oil production of superior quality from biochemical, nutritional and organoleptic points of view. In this regard, it was found that the higher expression of transcripts putatively associated with fatty acid biosynthesis and with the assembly of storage triacylglycerols was tightly linked with fatty acid accumulation pattern in olive fruits, starting at about 90 days after flowering until the end of fruit ripening. Moreover, transcripts related to the biosynthesis of structural proteins were correlated with the rapid cell divisions during fruit growth maintained the higher expression [18].

In this direction, a great-extended analysis between two different olive cultivars was performed by comparing the transcriptome of their drupes (cv. ‘Coratina’ with high conciseness in phenols and cv. ‘Tendellone’ with a lack of oleuropein natural variant) during two developmental stages, namely 45 and 135 days after flowering [19]. A foregoing SSR analysis indicated a very high genetic distance between the two cultivars and wondrous discrepancies in their biophenol accumulation pattern [27]. Data indicated that a percentage of 25% of the annotated transcripts that code enzymes were involved in lipids’ biosynthesis and metabolites of the fruit during ripening procedure, while transcript variations were fixed with drup’s physiological status [27]. In addition to this work, a ligament between geranylgeranyl reductase (CHL P) transcript levels and the concentration of tocopherols during olive pericarps’ maturation process was proposed [27]. Trying to examine the effect of ripening on the antioxidant capacity, *OeCHL P* transcript levels of eleven different olive cultivars were evaluated through qRT = PCR analysis at two developmental stages of the drupes [20]. The obtained data disclosed that the CHL P expression was remarkably induced during the ripening procedure in a cultivar-independent manner [20]. This study also showed that the concentration of phenols was reduced while the level of tocopherol was stimulated by ripening [20].

In order to decode flavonoid pathways across olive drupe development, the expression of related genes, including phenylalanine-ammonia-lyase (PAL), chalcone synthase (CHS), flavanone 3-hydroxylase (F3H), dihydroflavonol 4-reductase (DFR), anthocyanidin synthase (ANS), UDP-glucose-flavonoid 3-O-glucosyltransferase (UFGT), along with the concentration of flavonoid compounds and anthocyanins, was studied [21]. Flavonoid concentration increased in young fruit, whereas anthocyanins accumulated at ripening, in particular in epicarp tissue, concurrently with an up-regulation of UFGT. In addition, PAL, CHS, F3H and UFGT were up-regulated at the early stages of drupe development; DFR was induced in the epicarp at the onset of color change, while ANS transcripts were extremely abundant at a more advanced stage. These data suggested that DFR and ANS, together with UFGT, might represent key elements in the regulation of anthocyanin biosynthesis in olives [21].

Further research on this field was concentrated on the mRNA levels of 35 olive transcripts that were defined by RT-qPCR analysis in drupes from olive cultivars with a low and high concentration in phenolic compounds (‘Dolce d’Andria’ and ‘Coratina,’ respectively) across three different stages of fruit’s development [22]. Transcripts that participate in the biosynthesis of phenolics showed relative correlation with the concentrations of these compounds, indicating a transcriptional adjustment of the pathways that can be corresponded. Principally, *OeGES*, *OeDXS*, *OeADH* and *OeGE10H*, encoding geraniol synthase, 1-deoxy-D-xylulose-5-P synthase, arogenate dehydrogenase and geraniol 10-hydroxylase, respectively, were almost only observed in olive drupe at 45 days after flowering. These compounds are being suggested to have a major role during fruit maturation in arranging the accumulation of secoiridoids [22].

Another research on olive’s transcriptomics is focused on the de novo transcriptome reconstruction of olive fruits together with a full expression analysis between samples from ‘Leucocarpa,’ an olive variety characterized by a switch-off in skin color at full ripeness, and ‘Cassanese,’ used as control plant at 100 and 130 days after flowering using Illumina RNA-seq technology [23]. At this time point, 3792 and 3064 transcripts were, respectively, expressed in ‘Leucocarpa’ and ‘Cassanese’ genotypes in a different way, from a total of 103,359. Significant differences in flavonoid and anthocyanin transcript profiles, including chalcone isomerase (CHI), chalcone synthase (CHS), 4-coumarate-CoA ligase (4CL), cinnamate 4-hydroxylase (C4H), phenylalanine ammonia lyase (PAL) and anthocyanidin synthase (ANS), was emerged both during fruit maturation and in relation to genotypes [23]. Finally, transcripts abundance of MYB, MYC, and WD40-type TFs was higher in Cassanese cultivar than in Leucocarpa and was also directly related to anthocyanin accumulation [23].

### 3.2. Detecting Significant Changes in Protein Abundance during Olive Drupe Development

In general, proteomics can be described as the identification and quantification of the protein content of cells, tissues or organisms using analytical technologies. It is well established that fruit phenotypical alterations can be sufficiently described through changes in proteome rather than genome or transcriptome, as protein production and regulation are directly affected by numerous environmental factors. Consequently, proteomic analysis can offer an informative view regarding fruit’s response to various environmental stimuli and therefore contribute to ripening mechanisms [28]. The *Olea europaea* can be ranked as a non-model plant species, so olive research is facing a dearth of proteomic investigation. It is interesting to note that olive tissues (leaves, fruit) are difficult to handle during protein extraction due to their rich content in phenolic and lipid compounds that hinder the procedure and lead to low-quality extracts. It is suggested that the use of 10% trichloroacetic acid (TCA)/acetone in olive tissue powder prior to protein extraction can provide effective removal of organic and water-soluble contaminants and offer well-resolved 2-DE patterns [29,30]. Before 2010, several protein/enzyme studies on *Olea europaea* have been conducted, focusing on pollen allergens, seed storage, olive oil quality and stress responses [31] (Table 3). Though a great number of proteins are detected in olive samples, a small proportion of them is identified based on data about homologous sequenced organisms.

Several olive proteins exhibited tissue-specific accumulation patterns during drupe development; for example, thaumatin-like proteins were detected in the olive pulp while storage proteins, oleosins and histones were most abundant in seeds [32,33]. In the early stages of maturation, pit hardening procedures include high accumulations of seed storage proteins. The first report of olive seed storage proteins was by Wang et al. (2001) using SDS-PAGE, immunoblotting and N-terminal sequencing. Storage proteins were classified into the 11 S legumin family, and they seem to accumulate both in the endosperm and the cotyledons [34]. It was also stated that seed storage protein synthesis intensified alongside seed development, underlying tight genetic regulation of endosperm development. Furthermore, Zamora et al. (2001) studied the protein profile of drupes from two different olive cultivars (‘Picual’ and ‘Arbequina’) during maturation and pointed a 4.6 kDa polypeptide compound as one of the most significant constituents of olive mesocarp using both SDS-PAGE and amino acid analysis. Additionally, a positive correlation between oil and protein content during ripening was demonstrated [35]. Other protein-based studies focus on lipoxygenase content (LOX) due to its involvement in the formation of six-carbon aldehydes, responsible for the virgin olive oil aroma. LOX is a polypeptide with a molecular mass higher than 100 kDa found in olive fruit and, alongside other enzymes, such as alcohol dehydrogenase, fatty acid hydroperoxide lyase and polyphenol oxidase, are responsible for numerous organoleptic traits [36]. For instance, polyphenol oxidase and peroxidase could define the developmental stage of olive fruits as their concentrations seem to increase during maturity processes [37]. Another study also revealed that a β-glucosidase is involved in the transformation of the phenolic glucosides present in olive mesocarp, creating secondary metabolic compounds participating in olive drupe ripening [38].

Since comparative proteomic analysis can now be applied, a multitude of data regarding olive’s developmental stages or abscission is available in the bibliography (Table 3). Bianco and co-authors (2013) studied the proteome alterations correlated to olive (cv. ‘Coratina’) fruit ripening by means of comparative 2 DE-proteomics coupled with mass spectrometry analysis [39]. Data of this work indicated that 247 protein spots were altered across development, while 68 proteins were identified. In particular, proteins related to cell multiplication increased during the early fruit development while vacuolar ATPases, annexins and cytoskeletal proteins regulated cell expansion in later stages [39]. Information on several proteins associated with metabolism and fatty acids biosynthesis derives from unpublished gel-free proteomic work of our group on drupes of culti ‘Chondrolia Chalkidikis’ varvia nano-LC coupled to HDAM Orbitrap mass spectrometer. In this work, 3258 proteins were identified, from which the 350 were differentially accumulated between the two final maturation stages (green vs. black stage) of drupes. Additional research on olive fruit ripening processes, along with the complete cultivated olive genome sequence, will shed light upon processes that occur during olive maturation [40].

### 3.3. Olive Drupe ripening Trigger Fundamental Metabolic Alterations in Olive Drupes across Ripening

Ripening processes involve variations in the metabolic profile of drupes, mainly related to fatty acid and phenol biosynthesis. To date, using metabolomics technologies, several research groups are able to identify and monitor various metabolites that are present in the olive drupe-grown system, giving insights into its metabolism and physiology [42] (Table 4). In terms of structure and function, metabolites are grouped into primary and secondary metabolites [43]. Both can be assessed through a variety of analytical methods such as nuclear magnetic resonance (NMR) and gas or liquid chromatography, coupled with various mass spectrometry systems (GC-MS, LC-MS) (Table 4). All metabolic data generated are processed using online databases [44].

Olive oil’s beneficial health effects are highly correlated to its abundance in metabolites, especially phenolic compounds, such as tyrosol, hydroxytyrosol and oleuropein [45]. The metabolite profile of diverse olive tissues (i.e., leaves, roots, seeds and drupes) is available in the present day due to the recent advances in total metabolite extraction and identification. This knowledge can offer opportunities in comprehending plants’ responses under numerous conditions or interpret complex biological procedures, including drupe ripening. An applicable example is offered by Di Donna et al. (2010), who proposed that the determination of secondary metabolite profile in olive leaves could be used as a marker for cultivar differentiation [46]. Specifically, using HPLC combined with ESI MS approach, researchers identified 12 phenolic compounds, namely oleoside, verbascoside, oleuropein, lingstroside, hydroxytyrosol glucoside etc., in five cultivars (‘Carolea,’ ‘Cassanese,’ ‘Coratina,’ ‘Nocellara’ and ‘Leccino’). By applying two supervised pattern recognition applications, linear discriminant analysis and soft independent modeling of class analogy managed to predict effectively the cultivar and the cultivation zone of each sample [46].

Physiological responses of olive drupes in both abiotic and biotic stress conditions could be described through changes in metabolism; olive fruit tissues seem to produce metabolites involved in adaption/resistance to adverse conditions. For example, Martinelli et al. (2013), using GC-MS, detected 176 metabolites in olive drupes, of which 57 were water stress-related [47]. This work further indicated that 19 metabolites were negatively affected by water stress, including sugars, polyols and fatty acids. Similarly, Machado et al. (2013) concluded that irrigation during drupe maturation of cv. ‘Cobrancosa’ negatively influenced the olive oil quality retrieved due to decreases in phenylpropanoids, phenolic alcohols, secoiridoids and flavonoids content [48]. In another irrigation study, it was found that 46 mesocarp metabolites differed significantly in non-irrigated and irrigated cv. ‘Leccino’ trees, followed by alterations in the expression of genes related to polyphenol metabolic pathways such as chalcone synthase (CHS), phenylalanine ammonia lyase (PAL) and dihydroflavonol reductase (DFR) [24]. In particular, an increase in polyphenol and anthocyanin content was reported in non-irrigated fruits, followed by increased PAL activity in the early stages of development, possibly connected to stress-activated mechanisms during ripening. CHS and DFR transcripts accumulation increased mainly in epicarps of non-irrigated drupes during the late stages of maturation, serving in fruit pigmentation processes [24]. What is worth mentioning is that shikimic acid, galacturonic acid, allose and L-asparagine were increased dramatically in irrigated samples, pointing to a possible delay in drupe maturation processes related to higher irrigation levels [24].

The olive ripening procedure involves the activation of several metabolic pathways involving polyphenol and fatty acid production, which affects greatly the quality of the olive oil [56]. Trans, saturated, monosaturated and polysaturated fatty acid concentrations were strongly related to the ripening state of the drupe and indicated optimal harvest timepoint [49,50]. It has been demonstrated that the content of fatty acids significantly varied in various olive cultivars (‘Regarding ‘Arbequina,’ ‘Gordal,’ ‘Manzanilla,’ ‘Picual’ and ‘Picudo’), with cv. ‘Picudo’ exhibiting a continuous increase in total oil content across all ripening stages [49]. Another study identified, through HPLC/DAD/ESI/MS analysis, 20 different phenolic compounds during the ripening of three typical Tuscan cultivars, namely ‘Frantoio,’ ‘Moraiolo’ and ‘Leccino’ [50]. The presence of lignans and dialdehydic form of decarboxymethyl oleuropein aglycone (3,4-DHPEA-EDA) was excluded in all these fruits. In terms of total phenols, ‘Moraiolo’ was always the richest cultivar; the phenolic reduction was scarcely appreciable in ‘Frantoio,’ while for ‘Moraiolo’ and ‘Leccino,’ the decrease was almost inversely related to the ripening time [50]. The dynamics of squalene and sterols accumulation during olive maturation in the cultivars ‘Picual’ and ‘Arbequina’ as well as in two breeding selections derived from crosses between them were investigated [51]. This research revealed significant changes in squalene and sterols contents in fruits harvested from September to November but practically no differences between fruits harvested in November and December [51].

Modifications reported in drupe phenolic profile during maturation are strongly correlated to cultivar and define, to a major extent, the quality of oil extracted. For example, the phenolic content of various Spanish cultivars (‘Cornicabra,’ ‘Arbequina,’ ‘Picudo,’ ‘Morisca,’ ‘Picual’ and ‘Picolimon’) during fruit development was evaluated by Gomez-Rico et al. (2008) using an HPLC-MS [52]. During olive ripening, a decrease in oleuropein content in mature drupes of numerous cultivars has been reported; oleuropein steady-state level was negatively correlated to verbascoside content, which tends to highly accumulate in mature drupes [52,53]. It is well-established that these metabolites, alongside hydroxytyrosol, determine important organoleptic features of the drupes, such as their flavor [53]. Additionally, a great variety of phenolic compounds have been identified in olive drupes, displaying variations in content regarding the cultivar and maturity stage; this can lead to the categorization of cultivars into groups depending on their phenolic profile and, as a result, their quality [52]. In another study, ethanol was also quantified by HS-SPME-GC-FID in drupes of three olive cultivars (‘Picual,’ ‘Hojiblanca’ and ‘Arbequina’) during ripening, showing that its concentration increases rapidly near the harvest date (ethanol content varied between 0.56 and 59 mg/kg), and its synthesis is highly genetically regulated [54]. Ethanol accumulation intensified as mature olive drupes remained on the tree due to fruit anaerobic respiration. Lastly, variations in the content of pentacyclic triterpenoids during fruit maturation have been brought to light by using an HPLC-UV/vis analysis in drupes of two olive cultivars (‘Picual’ and ‘Cornezuelo’) [55]. Oleanolic acid and maslinic acid were identified in fruits, and their concentrations increased significantly during the early stages of fruit growth and development, demonstrating their role in drupe physiology, including its abiotic/biotic response [55].

The correlation of metabolome and transcriptional data on various olive tissues provides a comprehensive view of gene expression and metabolism that could enlighten the complex drupe maturity process. In this regard, Xiaoxia et al. (2020) combined metabolomics and transcriptomic analyses to characterize the interaction between gene expression and metabolite production in fruits, old and new leaves of the cultivar ‘Leccino’ [25]. In this analysis, 3049 newly annotated genes were identified; 2350 genes were functionally annotated, and the most up-regulated genes were related with fatty acid metabolism [25]. As distinct tissues, olive leaves and drupes reveal tissue-specific metabolic variations, as drupes synthesize a great number of fatty acids (oleic acid and palmitic acid being the most dominant), whereas leaves are characterized by an abundance of flavonoids and phenylpropanoids [57]. Recently, Bruno et al. (2019) applied a comparative transcriptomic analysis on fruits of the ‘Carolea’ cultivar trees planted at various altitude areas (10, 200 and 700 m) [26]. An important upregulation of FAD desaturation genes (FADs) specifically involved in the biosynthetic pathway of unsaturated fatty acids (FAs) along with downregulation of genes involved in secoiridoid formation was detected in drupes grown at the highest altitude (700 m). RNA-seq analysis revealed that *OeFAD2.2* and *FAD6* genes were upregulated in turning purple drupes growing in 700 m whilst *OeFAD7* gene was upregulated in drupes growing in 10 m altitude [26]. At lower altitude cultivation areas, phenol biosynthesis was enhanced while oleuropein production being higher in drupes growing in 700 m. These results suggested that stability of FAs and phenols, mainly of secoiridoids group, is promoted at high altitude, while at lower altitude phenol biosynthesis is prolonged [26].

## 4. Conclusions and Future Perspectives on Olive Ripening Studies

*Olea europaea* is one of the most important trees for the agricultural economy of the Mediterranean region. Its cultivation is now distributed worldwide along with the generation of new cultivars through breeding programs. Consequently, more scientists internationally tend to research olive plant physiology and drupe biology under numerous environmental conditions, with the aim of proposing future ways of sustaining olive production. Olive fruit ripening stages are generally characterized by significant changes in both morphology and biochemical content of the drupe, leading to the production of high or low-quality olive products. However, the complete mechanism of olive ripening is not yet understood due to its perplexing functioning and genotypic variations (cultivars). With olive cultivation being present since ancient times, it is expected that evolutionary time shaped its genome and, as a result, its physiology in a more complex way than other plant species. Epigenomic studies of olive ripening in numerous environments would provide answers regarding the aforementioned issue; however, no such attempt was recorded in recent literature. The available “-omics” technologies provide a precise and informative way to investigate such complicated biological phenomena as they offer holistic approaches. Recent technological advances in the “-omics” field have improved the means of extraction and identification and have generated a vast number of data that unveil novel gene expression and protein accumulation patterns or metabolic pathways. However, concerning olive biology investigation, there is still an inadequacy in genomics data, as the only available fully sequenced genome corresponds to the wild olive species (*Olea sylvestris*), including major differences in gene distribution and regulation. The complete curation of the cultivated *Olea europaea* genome sequence will aid researchers in their attempt to identify the gene expression alterations that result in olive drupe maturation. Once the full genome sequence becomes available, the known gene and proteins list will be enriched and evaluated by new entries, which will generate a final, holistic picture of olive fruit development.

## Figures and Tables

**Table 1:** Publicly available assemblies of olive genome.

Olive Variety	Genome ID	Genome Size (Gb)	Total Number of Scaffolds	Contig N50 (Kb)	Scaffold N50 (Kb)	Sequencing Platform(s)	Reference
‘Arbequina’		1.3	1290	4665	41,790	Nanopore & HiC	Rao et al., 2021 [14]
‘Farga’	OE6A	1.32	11,038	52	443	Illumina MiSeq	Cruz et al., 2016 [15]
sbsp. sylvestris	Oe451	1.46	41,256	25	228	Illumina HiSeq 2000	Unver et al., 2017 [16]
‘Picual’	Oleur061	1.68	8718	410	1145	PacBio RSII & Illumina HiSeq 2500	Jiménez-Ruiz et al., 2020 [17]

**Table 2:** Numerous transcriptomic studies investigated the transitions occurring in a molecular basis during drupe development.

Publication	Olive Cultivars		Ripening Stage		Plant Material	Approach
Alagna et al., 2009 [19]	‘Coratina’		45 DAF ^1^		fruit pulp	454 pyrosequencing
	‘Tendellone’		135 DAF			
Mazzalupo et al., 2011 [20]	Bardi i Tirana’	‘Nocellara del Belice’	129 DAF 190 DAF		pericarp	Quantitative Reverse—Transcription PCR
	‘Buscionetto’	‘Semidana’				(qRT-PCR)
	‘Carolea	‘Taggiasca’				
	‘Casalina’	‘Tonda Dolce’				
	‘Gaggiolo’	‘Verdello’				
	‘Gnagnaro’					
Martinelli et al., 2012 [21]	‘Leccino’		4 DAF	12 DAF	epicarp	RT-PCR
			9 DAF	16 DAF	mesocarp	Semi-quantitative PCR
Alagna et al., 2012 [22]	‘Coratina’	Dolce d’Andria’	45 DAF	120 DAF	fruit pulp	cDNA-AFLP analysis
	‘Rosciola’	‘Tendellone’	60 DAF	135 DAF		Semi-quantitative PCR RT-qPCR
	‘Frantoio’	‘Non Belice’	75 DAF	150 DAF		
	‘Canino’	‘Noc Etnea’	90 DAF	165 DAF		
	‘Moraiolo’	‘Bianchella’	105 DAF			
	‘Leccino’	‘Dritta’				
Iaria et al., 2016 [23]	‘Cassanese’		100 DAF		epicarp	Illumina HiSeq TM 2000
	‘Leucocarpa’		130 DAF		mesocarp	
Martinelli et al., 2012 [24]	‘Leccino’		DOY ^2^ 229	DOY 272	epicarp	RT-PCR
			DOY 255	DOY 302	mesocarp	
Xiaoxia et al., 2020 [25]	‘Leccino’		fully ripe		fruit pulp	Illumina HiSeq X10
					leaves	RT-Qpcr
Bruno et al., 2019 [26]	‘Carolea’		180 DAF		fruit pulp	Illumina HiSeq 2000
					oil	qRT-PCR

^1^ Days after flowering (DAF); ^2^ Day of the year (DOY).

**Table 3:** Proteomics analyses using gel or gel-free approaches demonstrated key features of olive ripening processes in previously published studies.

Publication	Olive Cultivars	Ripening Stage		Plant Material	Approach
Esteve et al., 2011 [32]	‘Picual’	fully ripe		fruit pulp	SDS-PAGE
	‘Frantoio’				MALDI-TOF MS
	‘Bent al Kadi’				nanoLC-MS/MS
	‘Mision de San Vicente’				
Esteve et al., 2011 [33]	‘Picual’	green-yellow		fruit pulp	SDS-PAGE
	‘Arbequina’	spotted			UHPLC
	‘Hojiblanca’	fully ripe			
	‘Gordal de Velez Rubio’				
	‘Manzanilla Cacerena’				
Wang et al., 2001 [34]	‘Picual’	fully ripe		germplasm	PAGE
					SDS-PAGE
					N-terminal sequencing
Zamora et al., 2001 [35]	‘Arbequina’	green		mesocarp	tricine-SDS-PAGE
	‘Picual’	spotted			HPLC
		fully ripe			
Ebrahimzadeh et al., 2002 [37]	‘Zard’	45 DAFS ^1^	120 DAFS	leaves	PAGE
		60 DAFS	135 DAFS	fruit pulp	SDS-PAGE
		75 DAFS	150 DAFS		
		90 DAFS	165 DAFS		
		105 DAFS	180 DAFS		
Bianco et al., 2013 [39]	‘Coratina’	45 DAF ^2^		epicarp	SDS-PAGE
		110 DAF		mesocarp	MALDI-TOF MS
		150 DAF			
Velázquez-Palmero et al., 2017 [41]	‘Arbequina’	63-133 DAF		endocarp	HPLC
	‘Picual’	161 DAF		mesocarp	SDS-PAGE
		196, 217 DAF		fruit pulp	Immunoblot analysis
		245 DAF			

^1^ Days after fruit set (DAFS). ^2^ Days after flowering (DAF).

**Table 4:** Analytical techniques that have been used in recent literature, providing a great amount of metabolomic data in order to interpret olive’s ripening complex regulation and physiology.

Publication	Olive Cultivars			Ripening Stage			Plant Material	Approach
Alagna et al., 2012 [22]	‘Coratina’	‘Dolce d’Andria’		45 DAF ^1^		120 DAF	fruit pulp	HPLC-DAD
	‘Rosciola’	‘Tendellone’		60 DAF		135 DAF		NMR
	‘Frantoio’	‘Non Belice’		75 DAF		150 DAF		
	‘Canino’	‘Noc Etnea’		90 DAF		165 DAF		
	‘Moraiolo’	‘Bianchella’		105 DAF				
	‘Leccino’	‘Dritta’						
Romero-Segura et al., 2012 [38]	‘Picual’			140 DAF		217 DAF	mesocarp	HPLC-MS-ESI
	‘Arbequina’			168 DAF		245 DAF	oil	
				196 DAF				
Martinelli et al., 2013 [47]	‘Cipressino’			236 DOY ^2^			epicarp	GC-MS
							mesocarp	
Machado et al., 2013 [48]	‘Cobrancosa’			fully ripe			fruit pulp	HPLC
Martinelli et al., 2012 [24]	‘Leccino’			DOY 229	DOY 272		epicarp	GC-MS
				DOY 255	DOY 302		mesocarp	
Gómez-González et al., 2011 [49]	‘Picual’	‘Gordal’		green		purple	oil	GC-MS
	‘Arbequina’	‘Picudo’		green-yellow		black		
	‘Manzanilla’			yellow-purple				
Cecchi et al., 2013 [50]	‘Frantoio’			105 to 189 DAF			fruit pulp	HPLC-DAD-ESI-MS
	‘Moraiolo’							
	‘Leccino’							
Fernandez-Cuesta et al., 2013 [51]	‘Picual’			fully ripe			epicarp	GC
	‘Arbequina’						mesocarp	NMR
Gómez-Rico et al., 2009 [52]	‘Arbequina’		‘Picolimon’	green			fruit pulp	HPLC-MS
	‘Cornicabra’		‘Picudo’	spotted			oil	GC-MS
	‘Morisca’		‘Picual’	black				
Dagdelen et al., 2013 [53]	‘Ayvalik’			fully ripe			fruit pulp	HPLC
	‘Domat’						oil	
	‘Gemlik’							
Beltran et al., 2015 [54]	‘Picual’			147 DAF		182 DAF	mesocarp	HS-SPME-GC-FID
	‘Hojiblanca’			154 DAF		210 DAF		
	‘Arbequina’							
Peragon et al., 2013 [55]	‘Picual’			green,		black skin & <50% purple flesh,	fruit pulp	HPLC-UV-Vis
	‘Cornezuelo’			green-yellow		black skin & >50% purple flesh,		HPLC-MS/MS
				spotted,		black skin & 100% purple flesh		
				reddish, brown,				
				black skin & white flesh				
Xiaoxia et al., 2020 [25]	‘Leccino’			fully ripe			fruit pulp	GC-MS
							leaves	
Bruno et al., 2019 [26]	‘Carolea’			180 DAF			fruit pulp	HPLC
							oil	GC-FID-MS

^1^ Days after flowering (DAF). ^2^ Day of the year (DOY).
